# Plasma p-tau217 and glucose metabolism correlate in neocortical association areas in Alzheimer's disease

**DOI:** 10.1093/braincomms/fcag074

**Published:** 2026-03-09

**Authors:** Lauren N Koenig, Hanna Huber, Anjalika Chongtham, Guglielmo Di Molfetta, Randolph D Andrews, Ana Lukic, Neeva Shafiian, Howard M Fillit, Kaj Blennow, Nicholas J Ashton, Henrik Zetterberg, Dawn C Matthews, Ana C Pereira

**Affiliations:** ADM Diagnostics, Inc., Northbrook, IL 60062, USA; Department of Psychiatry and Neurochemistry, Institute of Neuroscience and Physiology, Sahlgrenska Academy at the University of Gothenburg, 43180 Mölndal, Sweden; Department of Translational Dementia Research, German Center for Neurodegenerative Diseases (DZNE) Bonn, 53127 Bonn, Germany; Clinic of Old Age Psychiatry and Cognitive Disorders, University Hospital Bonn, 53127 Bonn, Germany; Department of Neurology, Icahn School of Medicine at Mount Sinai, NewYork, NY 10029, USA; Nash Family Department of Neuroscience, Friedman Brain Institute, Icahn School of Medicine at Mount Sinai, NewYork, NY 10029, USA; Department of Psychiatry and Neurochemistry, Institute of Neuroscience and Physiology, Sahlgrenska Academy at the University of Gothenburg, 43180 Mölndal, Sweden; ADM Diagnostics, Inc., Northbrook, IL 60062, USA; ADM Diagnostics, Inc., Northbrook, IL 60062, USA; Department of Neurology, Icahn School of Medicine at Mount Sinai, NewYork, NY 10029, USA; The Alzheimer's Drug Discovery Foundation, NewYork, NY 10019, USA; Department of Psychiatry and Neurochemistry, Institute of Neuroscience and Physiology, Sahlgrenska Academy at the University of Gothenburg, 43180 Mölndal, Sweden; Department of Psychiatry and Neurochemistry, Institute of Neuroscience and Physiology, Sahlgrenska Academy at the University of Gothenburg, 43180 Mölndal, Sweden; Banner Alzheimer's Institute, University of Arizona, Phoenix, AZ 85006, USA; Banner Sun Health Research Institute, Sun City, AZ 85351, USA; Department of Psychiatry and Neurochemistry, Institute of Neuroscience and Physiology, Sahlgrenska Academy at the University of Gothenburg, 43180 Mölndal, Sweden; Wisconsin Alzheimer's Disease Research Center, University of Wisconsin School of Medicine and Public Health, University of Wisconsin-Madison, Madison, WI 53792, USA; Department of Neurodegenerative Disease, UCL Institute of Neurology, London WC1N 3BG, UK; Clinical Neurochemistry Laboratory, Sahlgrenska University Hospital, Mölndal S-431 80, Sweden; Department and Laboratory Medicine, University of Wisconsin School of Medicine and Public Health, Madison, WI 53705-2281, USA; UK Dementia Research Institute at UCL, London WC1E 6BT, UK; Hong Kong Center for Neurodegenerative Diseases, InnoHK, Hong Kong, China; Centre for Brain Research, Indian Institute of Science, Bangalore 560012, Karnataka, India; ADM Diagnostics, Inc., Northbrook, IL 60062, USA; Department of Neurology, Icahn School of Medicine at Mount Sinai, NewYork, NY 10029, USA; Nash Family Department of Neuroscience, Friedman Brain Institute, Icahn School of Medicine at Mount Sinai, NewYork, NY 10029, USA; Sanford Grossman Interdisciplinary Program I Neural Circuitry and Immune Function, Icahn School of Medicine at Mount Sinai, NewYork, NY 10029, USA; Ronald M. Loeb Center for Alzheimer's Disease, Icahn School of Medicine at Mount Sinai, NewYork, NY 10029, USA

**Keywords:** Alzheimer’s disease, tau protein, plasma, FDG-PET, p-tau217

## Abstract

While the biomarkers available for Alzheimer's disease are continually expanding, including clinically approved blood tests, not all of the relationships between various biomarkers have been fully elucidated. In this study, we explore how regional brain metabolism, as measured by fludeoxyglucose-18 (FDG)-PET, relates to plasma biomarkers such as p-tau217, Glial fibrillary acidic protein (GFAP), phosphorylated-tau (p-tau)217/beta-amyloid (Aβ) 42, and Aβ42/40 in a cohort of participants with early symptomatic Alzheimer’s disease. P-tau217 showed a consistent pattern of participants with higher p-tau217 levels having increased hypometabolism in Alzheimer’s disease-related regions in neocortical association areas such as the lateral temporal cortex, the precuneus and inferior parietal cortex and atrophy accompanied by greater cognitive impairment. GFAP also related to regional hypometabolism and atrophy in regions known to be affected in Alzheimer’s disease, though with a slightly different regional pattern. We additionally observed that participants with equivalent biomarker levels still exhibited diverse patterns of FDG-PET and atrophy. This suggests that, despite the above correlations, imaging provides additional information. These findings support and extend our knowledge of how plasma p-tau217 relates to other Alzheimer’s disease biomarkers and cerebral metabolism, helping to contextualize both the benefits and limitations of these plasma biomarkers.

## Teaser

Koenig et al. report that p-tau217 and glial fibrillary acidic protein (GFAP) levels in plasma are linked to hypometabolism on [18F]Fluorodeoxyglucose PET and brain atrophy in neocortical association regions affected by Alzheimer’s disease. Variability in imaging despite similar biomarker profiles suggests that PET and volumetrics provide crucial complementary insights.

## Graphical Abstract

**Graphical Abstract:**
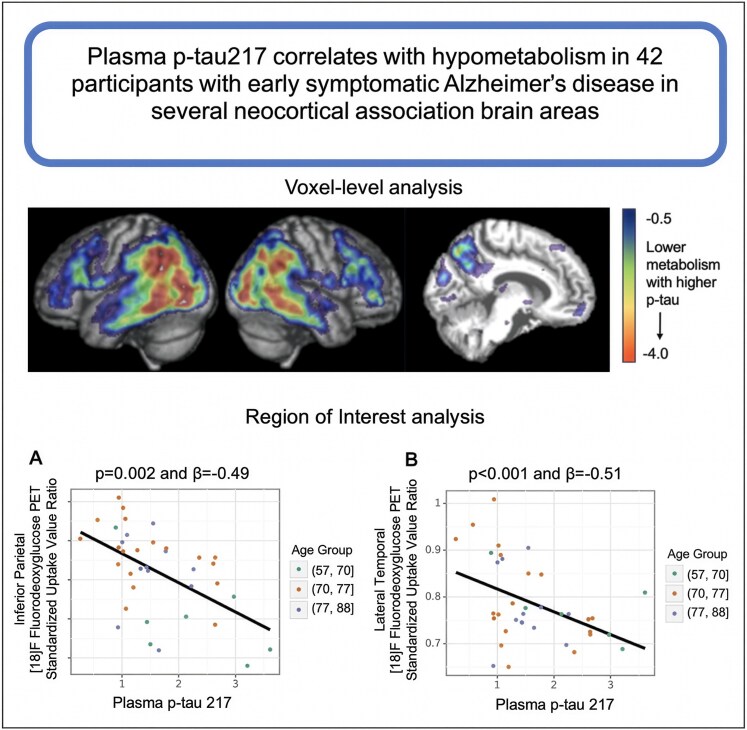

## Introduction

Alzheimer’s disease is the most common neurodegenerative disorder, characterized by the accumulation of amyloid plaques composed of beta-amyloid (Aβ) and neurofibrillary tangles formed of hyperphosphorylated tau.^1^ These pathological features are accompanied by neuroinflammatory and neurodegenerative processes that translate to progressive clinical dysfunction.^2^ Over the past decade, plasma biomarkers for Alzheimer's pathology have become a promising area of research and clinical use. These include the ratio of 42 to 40 amino acid-long Aβ (Aβ42/40),^3^ phosphorylated tau (p-tau), markers of glial activation (glial fibrillary acidic protein; GFAP),^4,5^ markers of neuronal damage (neurofilament light; NfL),^6^ and the ratio of p-tau217 to Aβ42, which is used in the Lumipulse blood test for the early detection of amyloid plaques associated with Alzheimer’s disease, recently approved by the U.S. Food and Drug Administration (FDA).^7^ Several p-tau markers, such as p-tau181, p-tau231, and p-tau217,^8-10^ have been found to be elevated in Alzheimer’s disease patients compared to age-matched controls and other neurodegenerative conditions.^11^ P-tau181, p-tau217, and GFAP have been demonstrated to increase with elevated amyloid burden^12^ and, in addition, have shown associations with tau-PET^13,14^ and post-mortem confirmed tau tangles.^15,16^ Aside from its correlations with amyloid and tau pathology, GFAP, a protein related to astrocyte reactivity in the brain, remains of high interest as a plasma biomarker in Alzheimer’s disease and related disorders to track astrogliosis.^4^ Among these plasma markers, p-tau217 has emerged as a particularly reliable biomarker^17-19^ for Alzheimer’s pathology, based on strong correlations with amyloid and tau-PET scans,^14,20-22^ post-mortem brain tissue analysis,^23^ and cerebrospinal fluid values.^17^

Data suggest that p-tau217 increases alongside amyloid in early disease stages and both tau and amyloid in later stages of disease,^23,24,^ although neurofibrillary tangles have been shown to be even more closely associated with alternate isoforms such as microtubule-binding region containing residue 243 (eMTBR-243).^25^ In a head-to-head study of p-tau plasma markers, p-tau217 was the best predictor of Alzheimer’s disease progression compared to plasma p-tau181, p-tau231, GFAP, and NfL.^23,26^ However, p-tau217’s relationships to regional brain function and volume, which also relate to tau accumulation, have not been fully explored.

Fluorodeoxyglucose (FDG)-PET, which measures regional cerebral metabolism through glucose uptake, the primary energy source in the brain, provides insight into brain function^27-29^ and has long been established to reflect regional changes associated with different dementias.^30^ In Alzheimer’s disease, disease progression correlates with a characteristic pattern of hypometabolism in areas that typically include the precuneus, parietal cortex, medial temporal lobe, posterior cingulate, and prefrontal cortex.^29,31,32^ This hypometabolic pattern is thought to possibly reflect the spread of tau pathology from the medial temporal lobe to the association neocortex, ultimately leading to neuronal dysfunction and loss.^33^ Notably, FDG-PET has been shown to strongly correlate with tau-PET measures^13,34,35^ and is associated with synaptic and neuronal loss.^36,37^ It is also well established that regional brain atrophy reflects progressive Alzheimer’s disease neurodegeneration, and correlates spatially with FDG-PET and tau-PET.^38-40^

The main objective of this study was to investigate the relationship between plasma biomarkers, specifically p-tau217, and regional cerebral metabolism as assessed by FDG-PET in participants with a clinical diagnosis of Alzheimer’s disease. We hypothesised that these participants, given clinical and FDG-PET profiles that were consistent with Alzheimer’s disease, would be in the ‘positive’ range for plasma p-tau217, suggestive of amyloid positivity,^20^ and that plasma p-tau217 values might increase with greater hypometabolism. Secondary objectives included exploring relationships of p-tau217 and other plasma biomarkers with regional MRI volumetrics and neuropsychological data, as well as examining the ratio of p-tau217 to Aβ42. This research sought to deepen our understanding of the role of p-tau217 in Alzheimer’s disease pathobiology and its relationship to specific neuroanatomical, functional and neurodegenerative effects.

## Materials and methods

### Study design

This exploratory study included participants from a pilot clinical trial of the drug riluzole and placebo in participants with early symptomatic Alzheimer’s disease.^41^ Participants were recruited from Rockefeller University Hospital and Icahn School of Medicine at Mount Sinai and had baseline blood draws, and the trial was approved by an Institutional Review Board (IRB) at both institutions. Participants underwent a neurologic and neuropsychological assessment, FDG-PET imaging, MRI imaging, and blood tests. Inclusion criteria included a clinical diagnosis of probable Alzheimer’s disease based on neurological and neuropsychological evaluation (National Institute on Aging-Alzheimer's Disease Association, NINCDS-ADRDA criteria), Mini-Mental State Examination (MMSE) score of 19 to 27, and age 50 to 95. The cognitive and functional performances of participants were staged using the Clinical Dementia Rating (CDR) scale for stratification, but were not used to determine inclusion. As part of the inclusion criteria, FDG-PET scans acquired at screening were examined visually by the local neuroradiologist for the presence of an AD-like pattern of glucose hypometabolism and the absence of patterns associated with other forms of dementia. One subject was excluded due to the neuroradiologist’s finding of a Lewy Body Disease (typically associated with occipital hypometabolism and a lack of posterior cingulate or hippocampal hypometabolism) or Parkinson’s Disease pattern without likely AD. Full inclusion/exclusion criteria and study design information can be found in Matthews *et al*.^41^ All neuroimaging was performed at Citigroup Biomedical Imaging at Weill Cornell Medicine under an IRB protocol separately approved by that institution.

All subjects from the clinical trial that had available data were included in this study, which led to a total of 42 participants. The available number (if less due to missing data) is specified for each analysis. Data from the baseline visit alone wereused to avoid drug-related effects.

### Fluid biomarkers

Blood collection used ethylenediaminetetraacetic acid (EDTA) tubes, which were immediately centrifuged at 1000 rpm at 4 °C, aliquoted into vials, and frozen at −80 °C. For Alzheimer’s disease-related plasma protein concentration measurements, the samples were thawed, centrifuged at 4000 g for 10 min at room temperature, and immediately prepared for measurement. Some samples may have been thawed and refrozen a few times, which could add additional variability to the measurements. The specific type of cryovial used is unknown. Plasma protein concentrations were measured employing the HD-X ultra-sensitive single molecule array (Simoa) platform (Quanterix, Billerica, Massachusetts, USA). NfL, GFAP, and Aβ42/40 concentrations were measured using the Neurology 4-Plex E kit (103670; Quanterix, Billerica, Massachusetts, USA); p-tau217 concentration was measured using the ALZpath kit (104371; Quanterix, Billerica, Massachusetts, USA) as previously described.^42,43^ Reagents from a single lot were used for the analysis of all samples. P-tau181 and p-tau231 were measured using in-house assays developed at the Clinical Neurochemistry Laboratory at the University of Gothenburg, Sweden, as previously described.^42,43^ Plasma samples were run in singlicates; calibrators and in-house plasma quality controls were run in duplicate. Assay repeatability/intermediate precision, based on control samples, was 3.6%/7.0% for NfL, 5.8%/8.7% for GFAP, 2.7%/3.9% for Aβ40, 3.3%/5.7% for Aβ42, 7.0%/10.2% for p-tau181, 8.3%/11.5% for p-tau231, and 4.8%/7.5% for p-tau217, respectively. All measurements were performed at the Clinical Neurochemistry Laboratory at the University of Gothenburg, Sweden.

The thresholds used for plasma p-tau217 were previously determined: p-tau217 positive was defined as > 0.63 pg/mL, negative as < 0.40 pg/mL, and intermediate as 0.40–0.63 pg/mL. The upper and lower bounds were selected such that a positive p-tau217 measurement indicates a 95% specificity for Aβ-positivity, while a negative measurement indicates a 95% sensitivity for Aβ-positivity.^20^

### Imaging biomarkers

For each FDG-PET scan, participants were administered 5 mCi of FDG followed by a 40-min uptake period; this period was without activity or audiovisual distraction, and the participant was in a resting state with eyes and ears open. Images were acquired on a Siemens Biograph 64mCT scanner as a series of four 5-minute frames; in some initial cases, these frames were extracted from a full dynamic scan. A T1-weighted volumetric MRI scan was also acquired using a spoiled gradient-recalled echo sequence (SPGR, repetition time 12.21 ms, echo time 5.18 ms, flip angle = 7°, voxels 0.94 × 0.94 × 1.5 mm) on a 3.0T GE SIGNA HDx system or a magnetisation-prepared rapid gradient-echo sequence (MPRAGE, repetition time 8.34 ms, echo time 1.7 ms, flip angle = 7°, voxels 0.94 × 0.94 × 1.5 mm) on a 3.0T GE Discovery MR750.

All PET and MRI images were visually inspected for apparent head motion and other quality issues. Motion correction was performed, and frames were averaged into a static image, which was co-registered to the participant’s T1-weighted MRI scan using Statistical Parametric Mapping Software (SPM12, Wellcome Trust). MRI scans were segmented into grey, white, and cerebrospinal fluid (CSF) tissue and spatially transformed to the SPM12 template, and the spatial transformations were then applied to the co-registered PET scans. Regions of interest were thresholded with a smoothed grey participant-specific grey matter segment, and average intensity within each region of interest was measured. Unlike the volumetric data, the measured regions focused on those previously shown to exhibit hypometabolism in AD. A reference region for calculation of standardized uptake value ratios (SUVRs) was defined based on paracentral voxels found to be preserved with the least variability in longitudinal FDG-PET progression studies.^41^ The pons was also measured as an alternate reference region for comparison and is explored in Supplementary Text 1. MRI scans were also segmented using FreeSurfer 6.0 with additional pre-processing and adjustment for intracranial volume, age, sex, and scanner as implemented in ADM Diagnostics’ CorInsights MRI® analysis tool, producing regional z-scores.

### Statistical analyses

The main analysis consisted of evaluating each plasma biomarker for relationships with 1) other plasma biomarkers, 2) FDG-PET SUVR in target brain regions, 3) cognitive test results, and 4) regional brain volumes in Alzheimer’s disease-relevant regions. Relationships to plasma biomarkers were assessed using general linear models with the log-transformed plasma biomarker as the independent variable (using Python 3.9.1 ‘statsmodels’ package ver. 0.14.0).^44^ The plasma biomarkers were log-10-transformed due to their non-normal distribution, as is common in the field. If a plasma marker was significantly related to both FDG-PET and volumetrics in the same brain region, an additional model was created to directly estimate the relationship between FDG-PET and volume.

The specific set of covariates included was individualized to each model, but may include age, years of education, sex, a combination of race and ethnicity, and Apolipoprotein E (*APOE)* ε4 carrier status. The covariates included in an individual model were determined by first evaluating a model that included all potential covariates in addition to the main variable of interest. Non-significant covariates were dropped from the model before refitting, and the process was repeated until any remaining covariates were significant. A significance threshold of *p* > 0.05 was used, although using *P* > 0.15 produced similar results. Which covariates were ultimately included for each model can be seen in Supplementary Table 1, where the standardized coefficient (*β*-weight) and *P*-value for each covariate are listed if included or left blank if not included. If no models contained a specific covariate, the corresponding column in Supplementary Table 1 is not present. The same information (*P*-value and *β*-value) is also listed for the main independent variable; it is these values that are described in the main results of the paper. Due to the exploratory nature of this work, the reported *P*-values have not been adjusted for multiple comparisons. Beyond the covariate approach described above, we attempted to dissociate the possible impacts of normative ageing and age-related disease differences on biomarker values. Alzheimer’s disease pathology and related effects on function and neurodegeneration have been shown to differ across the age range of this study, with greater frontoparietal effects observed in younger people.^45^ As this study contained no healthy controls, relationships to normal ageing were investigated through literature review and other in-house data sets. Based on these, normal ageing was expected to have negligible relationships with FDG-PET when using the paracentral reference region, a negligible effect on plasma *P*-tau217,^10,46-49^ a negligible or modest relationship to p-tau181,^10,50^ and significant relationships with regional brain volume and with plasma GFAP and NfL. Volumes were pre-adjusted for age, sex, and scanner using CorInsights software to minimize non-disease related volumetric effects. For GFAP and NfL, supplemental analyses were performed using in-house normal ageing data (on which the same assays had been used) to investigate effects (Supplementary Text 2). Relationships between biomarkers were also examined within age-based subgroups to assess consistency across age. Prior studies have suggested that sex can be a significant covariate in brain volume, and GFAP values may be elevated in females.^51^ However, the pre-adjustment of volume accounted for volumetric sex differences, and the inclusion of sex as a covariate accounted for potential GFAP effects.

Given the relatively small available sample size, it was noted that the presence of outliers or a change in the statistical setup might lead to different results than those reported for all available data. To better quantify the uncertainty surrounding our findings, we used a bootstrapping approach to further examine the relationships that the previous models had found to be significant (independent coefficient having a *P*-value < 0.05). For each model, we randomly selected subjects with replacement—which allows for an iteration to include a particular subject multiple times or not at all—until reaching a cohort of the same size as the original number of subjects. This new cohort was modelled in the same way, and the process was repeated 1000 times. This iterative process provided a distribution of results from the model, showing how consistent our results were when varying the participants included, and was used to generate 95% confidence intervals.

For the purposes of exploring whether there was a pattern of hypometabolism associated with plasma p-tau217 values, and consistency with regional findings, the spatially transformed FDG-PET scans were also evaluated using a voxel-based approach. Images were grouped into four classes of approximately equal size based on plasma p-tau217 level. These were input into nonparametric prediction, activation, influence, and reproducibility resampling (NPAIRS) machine learning classification software to generate up to three (N-1) patterns (Canonical Variates) of hypometabolism and metabolic preservation that accounted for differences between groups. In brief, NPAIRS first performs feature reduction using Principal Component Analysis (PCA) and then combines the PCs into Canonical Variates, a form of linear discriminant analysis. It does so while repeatedly splitting the data set into halves and each time comparing the models developed for each data set half for reproducibility (correlation between the two models) and prediction (prediction of one half using the other half), resulting in consensus patterns that minimize overfitting.^52^ Each participant receives numeric scores quantifying their pattern expression.

Finally, we also qualitatively examined FDG-PET and volumetric results on an individual basis of participants having similar and dissimilar plasma p-tau217 levels to better understand the potential contribution of each biomarker to overall clinical assessment.

## Results

### Patient characteristics

In the original clinical trial, a total of 94 participants were screened at the two performance sites, of which 44 did not meet the inclusion/exclusion criteria. Of the 50 participants who were a part of the trial, 42, all of whom had a clinical diagnosis of Alzheimer’s disease, had data that allowed for inclusion in at least some of the analyses of this study. While missing data varied for each biomarker and clinical endpoint, all 42 had FDG-PET imaging biomarkers available, and 37 had plasma p-tau217 measurements available. A diverse range of age, clinical, and biomarker characteristics were found in this population, as in other Alzheimer’s disease studies.^41,53^ Participants had an average age of 74.6 years (standard deviation 7.0, range 58 to 88), 62% were women, and 59% were *APOE* ε4 carriers (Table 1).

**Table 1:** Demographics

Variable	Mean and standard deviation
n	42
Age (years)	74.6 (SD 7.0)
Education (years)	15.5 (SD 2.9)
Sex	Men: 16 (38%) Women: 26 (62%)
Race & Ethnicity	Black: 2 (5%) Hispanic/Latino: 0 (0%) White & non-Hispanic: 36 (95%)
*APOE* ε4 carrier	Yes: 22 (59%) No: 15 (41%)
P-tau217 (pg/mL)	1.6 (SD 0.79)
P-tau181 (pg/mL)	9.5 (SD 5.3)
P-tau231 (pg/mL)	27 (SD 19)
P-tau217/Aβ42	0.40 (SD 0.24)
Aβ42/40	0.07 (SD 0.01)
GFAP (pg/mL)	232 (SD 96)
NFL (pg/mL)	37 (SD 15)

Abbreviations: *APOE* = Apolipoprotein E, SD = standard deviation, NFL = neurofilament light, GFAP = glial fibrillary acidic protein.

The characteristics in the top half of Table 1 describe the variables considered for inclusion as covariates in each model. Most models did not have any covariates pass our selection process. When at least one covariate was selected for inclusion, the most common covariate was age, which was included in 100 of the 630 models. Less common were years of education in 29 models, sex in 8 models, and *APOE* ε4 status in 3 models. The combination of race and ethnicity was not found to be significant in any model (Supplemental Table 1). The covariates included in a specific model can be seen in Supplemental Table 1, where p and β values are listed when the model included that specific covariate, and left blank if that covariate was not included.

Disease severity was variable, with MMSE scores varying from 19 to 27 (mean 23, standard deviation 2.8). Hippocampal atrophy *z*-scores ranged from −5.75 to 0.73 in the left hemisphere and −5.04 to −1.72 in the right hemisphere, and inferior parietal atrophy *z-scores* ranged from −3.6 to 2.31 in the left hemisphere and −4.77 to 2.48 in the right hemisphere. Inferior parietal hypometabolism SUVR ranged from 1.6 to 1.0 (or 0.79 to 1.54 if referenced to pons), and lateral temporal hypometabolism SUVR ranged from 0.6 to 1.0 (or 0.87 to 1.48 if referenced to pons).

### P-tau217

**Figure 1:**
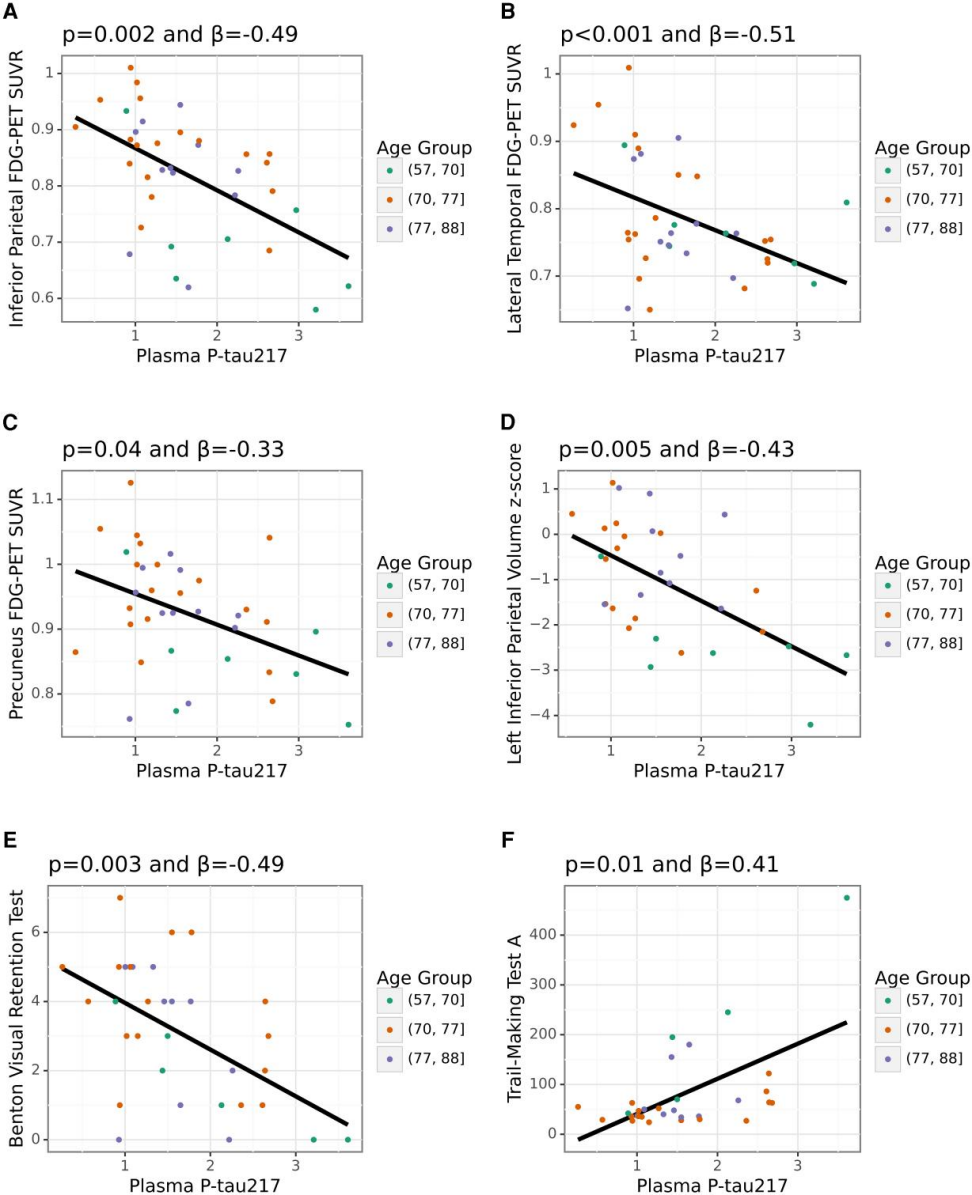
**Relationships found for plasma p-tau217.** (A–C) P-tau217 relationships with FDG-PET. **(D)** P-tau217 relationships with volumetric studies. **(E–F)** P-trau217 relationships with cognitive testing. Individual participants’ points are coloured based on age to qualitatively assess possible age-related differences, as the remaining sample size in each age subgroup precluded statistical analysis. The age ranges used are a balance of having equal numbers of participants in each subgroup (ages 57–74, 74–77, 77–88) versus having the range of ages in each subgroup be equal (ages 57–68, 68–78, 78–88). A black trend line is included to help to indicate the relationship, and plasma p-tau217 is displayed in its raw (not log-10-transformed) format. The *P*-value and *β*-value displayed in the title for each subgraph relate to the main analyses, which use the log-10-transformed plasma data and may include covariates in the general linear model (covariates were included only if significant). Abbreviations: FDG-PET = [18F]Fluorodeoxyglucose positron emission tomography, SUVR = Standardized Uptake Value Ratio.

Plasma p-tau217 was inversely related to regional cerebral glucose metabolism, such that higher levels of p-tau217 corresponded to reduced metabolism in the inferior parietal cortex (*β* = −0.49, *P* = 0.002), lateral temporal cortex (*β* = −0.51, *P* = 0.001), and precuneus (*β* = −0.33, *P* = 0.04). These relationships, involving neocortical association regions critical for integrating higher cognitive functions, are illustrated in Fig. 1A-C and Table 2. An inverse relationship was also observed with the sensorimotor cortex (*β* = −0.35, *P* = 0.03) (Table 2). To determine the extent to which individual subjects influenced our results, the median and 95% confidence intervals from the bootstrapped replicates were also measured: inferior parietal cortex (*β* = −0.49, [−0.85 to −0.26]), lateral temporal cortex (*β* = −0.51, [−0.77 to −0.22]), precuneus (*β* = −0.33, [−0.80 to 0.01]), and sensorimotor cortex (*β* = −0.35, [−0.67 to 0.12]) (Supplementary Fig. 2). A significant relationship was not observed between plasma p-tau217 and medial temporal metabolism.

**Table 2:** Key significant results for P-tau217

Variable	*P-value*	*β*-value	# of participants	type
Benton Visual Retention Test	0.003	−0.49	33	Cognitive
Digit Span Test	0.04	−0.35	34	Cognitive
Trail-Making Test A	0.01	0.41	31	Cognitive
Inferior Parietal FDG-PET SUVR	0.002	−0.49	38	FDG-PET
Lateral Temporal FDG-PET SUVR	<0.001	−0.51	38	FDG-PET
Precuneus FDG-PET SUVR	0.045	−0.33	38	FDG-PET
Sensorimotor FDG-PET SUVR	0.03	−0.35	38	FDG-PET
Plasma GFAP (log10)	0.01	0.40	39	Plasma
Plasma P-tau181 (log10)	0.004	0.45	39	Plasma
Plasma P-tau231 (log10)	0.03	0.36	36	Plasma
Left Inferior Parietal Volume z-score	0.005	−0.43	32	Volumetric
Right Inferior Parietal Volume z-score	0.04	−0.37	32	Volumetric
Right Lateral Ventricles Volume z-score	0.03	0.39	32	Volumetric

Abbreviations: FDG-PET = [18F]Fluorodeoxyglucose Positron Emission Tomography, GFAP = glial fibrillary acidic protein, SUVR = standardized uptake value ratios.

**Figure 2:**
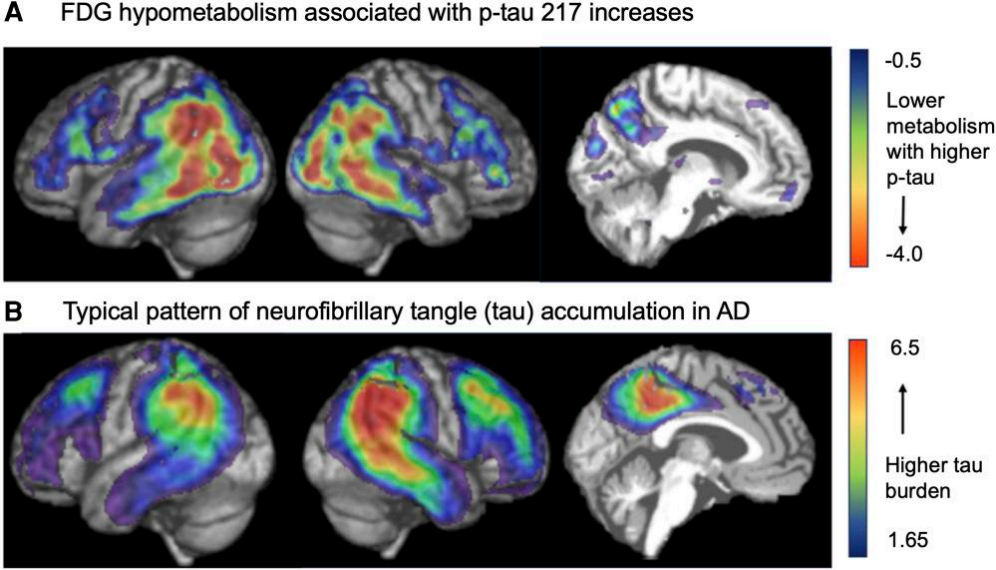
**Voxel-level relationship between p-tau217 and FDG-PET. (A)** The association of plasma p-tau217 with FDG-PET in this cohort at the voxel level (n = 35). Individual voxels are coloured based on the strength of the relationship found between plasma p-tau217 and FDG-PET SUVR. Relationships were determined using a multivariate classifier with classes defined by increasing p-tau217 levels. Values correspond to the first Canonical Variate, which represents the weight (unitless) that the NPAIRS model gives each voxel when predicting the level of p-tau217. **(B)** The pattern of tau-PET is typically seen in AD. Included to illustrate the strong similarity between the two patterns. This pattern was developed using spatially normalized, intensity normalized tau-PET scans from an Alzheimer’s disease cohort^39^ (n = 38), grouped by increasing Standardized Uptake Value Ratio. Values correspond to the first Canonical Variate, which represents the weight the NPAIRS model gives each voxel when predicting the level of p-tau217 (unitless). Abbreviations: AD = Alzheimer’s disease, FDG-PET = [18F]Fluorodeoxyglucose positron emission tomography, SUVR = Standardized Uptake Value Ratio, NPAIRS = nonparametric prediction, activation, influence, and reproducibility resampling.

All of these relationships remained significant (or not significant) when using the pons as a comparative reference region, except for the precuneus, which lost significance (Supplementary Text 1). When evaluated on a voxel-level basis, increasing plasma p-tau217 levels were associated with the expression of a pattern of hypometabolism in inferior parietal cortex, precuneus, and lateral temporal region, consistent with regional findings (Fig. 2). As with regional relationships, individual values vary within this general association.

Plasma p-tau217 levels were also negatively associated with grey matter volume in several regions, including the inferior parietal cortex (left *β* = −0.43, *P* = 0.005, right *β* = −0.37, *P* = 0.04) as shown in Fig. 1D and Table 2. Additional combination regions that included the inferior parietal volume or its individual gyri (angular gyrus and supramarginal gyrus) were also significant (Supplementary Table 1). Consistent with these findings, inferior parietal hypometabolism from FDG-PET was associated with reductions in both left and right inferior parietal volume (left *β* = 0.60, *P* < 0.001, right *β* = 0.56, *P* < 0.001) (Supplementary Table 2). A positive association was also observed with increased volume of the right lateral ventricle (*β* = 0.39, *P* = 0.027), suggesting a further relationship to structural changes typically observed in Alzheimer’s disease (Table 2).

P-tau217 was also associated with performance on several cognitive tests. Specifically, we observed negative relationships with the Benton Retention Test (a visuo-perceptual memory test) (*β* = −0.49, *P* = 0.003, Fig. 1E), the Trail-Making Test A (which assesses complex attention, visual-conceptual abilities, and visual-motor tracking) (*β* = 0.41, *P* = 0.01, Fig. 1F), and the Digit Span Test (*β* = 0.35, *P* = 0.045) (Table 2).

Using the thresholds the performing laboratory had previously determined for this assay, 96% of participants were considered positive for plasma p-tau217, indicating a high likelihood of amyloid positivity.^20^ One participant (2% of the cohort) had an intermediate measure of p-tau 217 (0.57 pg/mL, close to the 0.63 pg/mL positive threshold); they had an early symptomatic Alzheimer’s disease-like pattern of hypometabolism and exhibited a pattern of temporoparietal atrophy on MRI. Only one (2% of the cohort) was considered p-tau217 negative (at 0.27 pg/mL), with ‘negative’ in this case indicating a high likelihood of amyloid negativity. This participant exhibited an asymmetric pattern of precuneus, inferior parietal, and hippocampal hypometabolism, as well as profound asymmetric hippocampal and parietal atrophy and ventricular enlargement.

While the relationships between plasma p-tau217 and imaging biomarkers were significant, the individual points in the plots of Fig. 1 indicate the substantial variability of p-tau217.^23,40^  Supplementary Fig. 1 has example images of volumetric atrophy for individual participants across a range of plasma p-tau217 values. Those having near-identical plasma p-tau217 values exhibit diverse spatial distribution and severity of volume loss. This was true for both older participants and younger participants, who are less likely to have comorbidities.

The most significant correlations observed for ptau-217 were also seen for the ratio of ptau-217 to Aβ42: the ratio continued to be associated with parietal and temporal FDG-PET uptake, though the association (slope) is slightly weaker in the ratio versus p-tau217 by itself. Several variables that showed significant associations with p-tau 217 were not significantly related to the ratio, including FDG-PET uptake in the precuneus and sensorimotor regions, plasma GFAP, Digit Span Test, and several volume measurements (primarily parietal and ventricular volumes). Conversely, a few significant relationships are only observed with the ratio and not with p-tau217 alone: plasma Aβ40 and Aβ42, as expected, along with several volumes: insula, middle temporal gyrus, and anterior cingulate cortex.

### Comparisons of P-tau epitopes

In this cohort, p-tau217 was associated with p-tau181 (*β* = 0.45, *P* = 0.004) and, to a lesser extent, with p-tau231 (*β* = 0.36, *P* = 0.03), while p-tau181 demonstrated a robust relationship with p-tau231 (*β* = 0.90, *P* < 0.001) (Supplementary Fig. 3, Supplementary Table 1). The association between p-tau181 and p-tau231, which was consistent across age groups, was notably stronger than either biomarker’s relationship to p-tau217. The milder association of p-tau217 with the other two isoforms can be seen in Supplementary Fig. 3, where the spread of points is greater around the trend line. Specific points can be observed to have relatively large values for p-tau217 while having relatively small values for p-tau181 or p-tau231, as well as the reverse pattern.

The disparities between p-tau217 and the other two p-tau markers translated into differences in their relationships with FDG-PET. No significant relationships were identified between these markers and FDG-PET in parietal, precuneus, or temporal regions. Positive relationships were observed between p-tau181 and p-tau231 with medial temporal glucose metabolism (*β* = 0.55, *P* < 0.001; *β* = 0.35, *P* = 0.03, respectively) that were not observed with p-tau217. These relationships appeared consistent when stratified by age, CDR, plasma Aβ42/40, and plasma GFAP levels (Supplementary Fig. 4 and Supplementary Table 3; not evaluated statistically due to small subgroup size). Additionally, we observed positive relations between these p-tau markers and volumetrics in multiple brain regions, which were in the opposite direction to the patterns observed with p-tau217 (Supplementary Table 1). While these are preliminarily examined in the supplement, they warrant further investigation to clarify the implications of these unexpected associations.

### Findings for GFAP, NfL, and Aβ42/40

**Figure 3:**
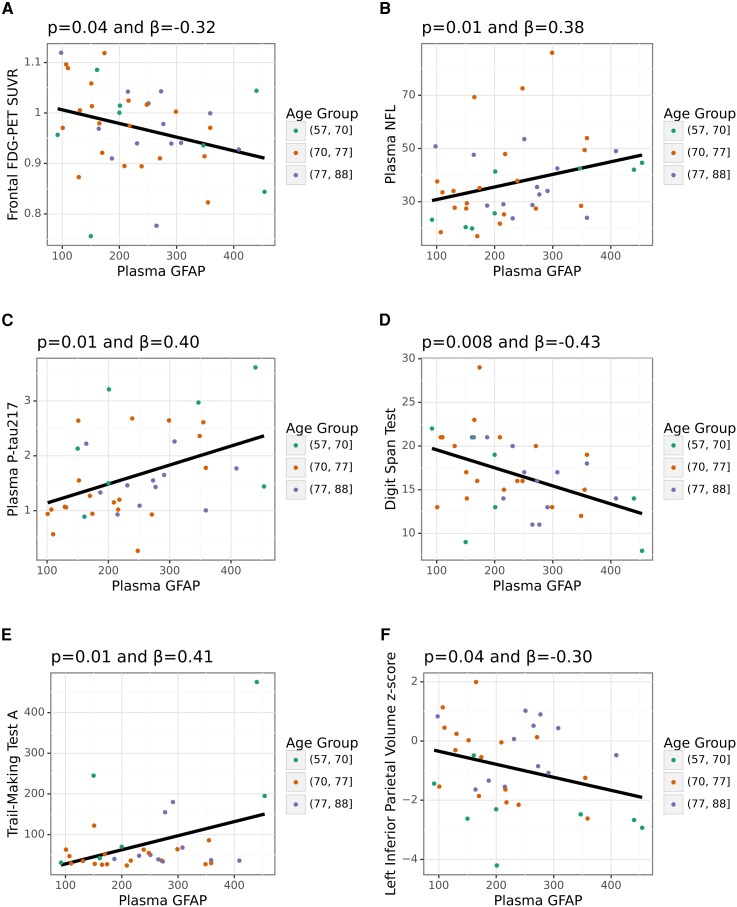
**Relationships found for plasma GFAP. (A)** GFAP relationships with FDG-PET. **(B)** GFAP relationship with NfL. **(C)** GFAP relationship with p-tau217. **(D-E)** GFAP relationship with cognitive testing. **(F)** GFAP relationship with cognitive testing. Individual participants’ points are coloured based on age to qualitatively assess possible age-related differences, as the remaining sample size in each age subgroup precluded statistical analysis. A black trend line is included to help to indicate the relationship, and plasma GFAP is displayed in its raw (not log-10-transformed) format. The *P-*value and *β*-value displayed in the title for each subgraph relate to the main analyses, which use the log-10-transformed plasma data and may include covariates in the general linear model (covariates were included only if significant). Abbreviations: FDG-PET = [18F]Fluorodeoxyglucose positron emission tomography, GFAP = Glial fibrillary acidic protein, NfL = neurofilament light, SUVR = Standardized Uptake Value Ratio.

We found a negative relationship between GFAP levels and FDG-PET measures of glucose metabolism in the frontal cortex (*β* = −0.32, *P* = 0.04, Fig. 3A). Plasma GFAP levels were positively associated with plasma NfL (*β* = 0.38, *P* = 0.01) and p-tau217 (*β* = 0.40, *P* = 0.01), and with the cognitive measures Digit Span Test (*β* = −0.43, *P* = 0.008), Trail-Making Test A (*β* = 0.41, *P* = 0.01), and Trail-Making Test B (*β* = 0.50, *P* = 0.03) (Fig. 3, Table 3).

**Table 3:** Key significant results for GFAP

Variable	*P-value*	*β*-value	# of participants	type
Digit Span Test	0.008	−0.43	37	Cognitive
Trail-Making Test A	0.01	0.41	34	Cognitive
Trail-Making Test B	0.03	0.50	19	Cognitive
Frontal FDG-PET SUVR	0.04	−0.32	42	FDG-PET
Plasma NFL (log10)	0.01	0.38	44	Plasma
Plasma P-tau217 (log10)	0.01	0.40	39	Plasma
Left Inferior Parietal Volume z-score	0.04	−0.30	36	Volumetric
Left Parahippocampal Volume z-score	0.03	0.37	36	Volumetric
Right Hippocampal Volume z-score	0.046	0.34	36	Volumetric
Right Pericalcarine Volume z-score	0.03	0.37	36	Volumetric

Abbreviations: FDG-PET = [18F]Fluorodeoxyglucose Positron Emission Tomography, GFAP = glial fibrillary acidic protein, NFL = neurofilament light, SUVR = standardized uptake value ratios.

In line with regional metabolic changes, GFAP levels also showed a negative relationship with regional MRI volumetrics in areas such as the combined left inferior parietal cortex and supramarginal gyrus (*β* = −0.30, *P* = 0.04) (Fig. 3, Table 3). However, we also observed relationships in the reverse direction in the hippocampus (*β* = 0.34, *P* = 0.046), parahippocampus (*β* = 0.37, *P* = 0.03), and pericalcarine cortex (*β* = 0.37, *P* = 0.03) (Table 3). These relationships remained even when using age-adjusted GFAP data (Supplementary Text 2).

In addition to NfL’s relationship with GFAP, NfL was also related to plasma p-tau181 (0.03, *β* = 0.33), right putamen volume z-score (*β* = −0.36, *P* = 0.04), and left hippocampal volume z-score (*β* = 0.33, *P* < 0.001) (Supplementary Table 1).

For the Aβ42/40 ratio, we observed a positive relationship with FDG-PET measurements in the precuneus (*β* = 0.37, *P* = 0.02) and medial temporal lobe (*β* = 0.39, *P* = 0.01) (Supplementary Table 1). As a lower Aβ42/40 ratio in plasma corresponds to higher values of amyloid-PET in Alzheimer’s disease,^3^ these relationships link lower metabolism to greater Aβ deposition in the brain. We also found a positive relationship between the plasma Aβ42/40 ratio and grey matter volume in the inferior parietal cortex (*β* = 0.39, *P* = 0.02). The relationship to plasma p-tau217 was not significant (*β* = 0.21, *P* = 0.25).

## Discussion

### P-tau217

The primary finding of this exploratory study in early symptomatic Alzheimer’s disease is that plasma p-tau217 is associated with glucose hypometabolism in Alzheimer’s disease-relevant regions in neocortical association areas, consistent with our hypothesis. Plasma p-tau217 levels showed negative correlations with FDG-PET in the inferior parietal cortex, lateral temporal cortex, and precuneus, which are critical for higher cognitive function and the integration of information. The inferior parietal cortex and lateral temporal cortex had the strongest associations, while the precuneus lost significance when analysed with our alternative reference region, the pons. We additionally observed a negative association with metabolism in the sensorimotor cortex, which we hypothesize is driven by late-stage tau pathology or corticobasal syndrome due to Alzheimer's disease (Supplementary Fig. 1).

These findings were consistent with the negative relationship observed between plasma p-tau217 and grey matter volume, as was seen in the inferior parietal cortex, which includes both angular and supramarginal gyri. These relationships with neocortical functional loss and atrophy are noteworthy as, while amyloid pathology accumulates across the neocortex early in the disease course, tau pathology typically starts in the entorhinal cortex and hippocampus and expands its accumulation to affect these cortical association areas as disease progresses.^38,54,55^ This progression leads to neuronal dysfunction and, over time, neuronal loss and associated cognitive impairment. The relationships we observed between higher p-tau217 and worse performance on several cognitive measures further support this link.

Previous research has suggested that, among the plasma biomarkers evaluated in this study, p-tau217 is the most reliable predictor of cognitive decline in Alzheimer’s disease.^23^ Prior work has also demonstrated that p-tau217 outperforms other p-tau markers when comparing Area Under the Curve (AUC) with amyloid and tau-PET imaging.^11,20,22^ This study offers additional support for plasma p-tau217 as a key biomarker associated with regional cerebral glucose metabolism and volumetric decline, particularly in neocortical association regions that are prominently affected by Alzheimer’s disease pathology. Our findings also show that plasma p-tau217 and imaging biomarkers provide important synergistic information to characterize a person’s disease status. While p-tau217 was associated with functional and neuronal loss, there was notable dispersion similar to what has been seen in published correlations of p-tau217 with tau-PET.^24,50^ The imaging data provided insight regarding the spatial distribution and severity of functional and neurodegenerative effects (Supplementary Fig. 1 subplot B). This information can help to assess whether neurodegeneration is likely responsible for clinical effects, identify comorbid conditions, and potentially predict the distribution of neurofibrillary tangles. Finally, although the p-tau217/Aβ42 ratio showed a largely similar pattern of associations as p-tau217 alone, the reduced or eliminated correlations suggest it also introduced additional variability. The ratio may be more useful in cohorts with a broader range of amyloid levels rather than in cohorts like the one used in this study, which are expected to be primarily amyloid positive.

### Comparisons of P-tau epitopes

In our analysis, plasma p-tau217 showed a stronger association with p-tau181 than with p-tau231. Notably, p-tau181 was robustly related to p-tau231, and neither was as strongly related to p-tau217 as we initially expected.

The positive associations of p-tau181 and p-tau231 with medial temporal FDG-PET and volume, concomitant with a lack of relationship to other Alzheimer’s disease-related regions, were also unexpected and were inconsistent with p-tau217 findings. These positive associations additionally were not seen in a prior study of the relationship between ptau-181, FDG-PET, and volume in the context of Alzheimer’s disease.^13^

Several studies have assessed the association between plasma p-tau biomarkers. Bayoumy *et al*. found that plasma levels of p-tau181 and p-tau231 were strongly correlated across most high-sensitivity assays (Spearman’s rho > 0.86),^56^ while others found a moderate correlation (*r* = 0.64, *P* < 0.0001)^57^ or moderate concordance (73%),^58^ reflecting overlapping but distinct roles of these tau species. Some studies reporting a tighter correlation between p-tau217 and p-tau181 have included larger proportions of amyloid-negative participants that serve as a low-end anchor and strengthen correlation slopes.^50^ When restricted to primarily amyloid-positive data (as in our study), those studies show dispersion between the plasma biomarkers and differing correlations with tau-PET.^50^ Studies comparing p-tau assays often focus on a binomial positive/negative comparison rather than the correlation within the positive range.^39^

Variations in inter-marker relationships and diagnostic performance are also likely influenced by differences in pathological stages, disparity in what the p-tau species are measuring,^59^ and differing responses to ageing processes and diverse clinical presentations of Alzheimer’s disease across different age groups.

### GFAP, NfL, and Aβ42/40

While GFAP has a variety of functions in the brain, in the context of Alzheimer’s disease, it is generally interpreted as a marker of astrocytic activity and neuroinflammation. GFAP levels are elevated in regions surrounding Aβ plaques and increase with tau accumulation in Alzheimer’s disease brains, indicating their relevance to both amyloid and tau pathologies.^60-62^ However, plasma GFAP correlates more strongly with cerebral Aβ pathology^4^ than with tau, suggesting that astrocyte activation may be more responsive to amyloid-driven pathology in the early stages of Alzheimer’s disease. Our findings suggest that elevated GFAP is also implicated in cellular damage, tau pathology and cognitive decline, as we observed a positive relationship between GFAP and NfL, a marker of axonal injury, as well as with p-tau217 levels and general cognitive measures such as the Digit Span Test, Trail-Making Test A, and Trail-Making Test B. These results align with previous studies linking elevated GFAP to cognitive decline in Alzheimer’s disease and the conversion from mild cognitive impairment to Alzheimer’s disease,^12,63,64^ highlighting astrocyte reactivity as a contributor to neuronal damage and disease progression.

Consistent with these relationships, plasma GFAP levels were negatively related to grey matter volume in the inferior parietal cortex as well as with FDG-PET measures of glucose metabolism in the frontal cortex. The negative relationships overlap with previous findings in a separate Alzheimer’s disease cohort that observed negative relationships in lateral temporal and middle frontal regions^13^ and with the observed cognitive and plasma NfL results. However, we also found positive relationships between GFAP and brain volume in the hippocampus and parahippocampus, which remained even when attempting to adjust for normal ageing effects. Previous studies looking at the association between plasma GFAP levels and brain volumetrics have shown varying results,^65,66^ and the relationships with medial temporal regions require further investigation.

In addition to GFAP, NfL was also significantly associated with p-tau181 and several brain volumes, but in general had fewer significant associations than other plasma markers. Additionally, no strong association was observed with FDG uptake, nor with p-tau217, which deviates from prior literature.^13^

We found a positive relationship between the plasma Aβ42/40 ratio and FDG-PET measures in both the precuneus and medial temporal lobe areas typically affected early in Alzheimer’s disease progression, similar to what has been seen previously.^67^ Aβ42/40 did not relate to plasma p-tau217. Plasma Aβ42/40 has been reported to correlate only moderately with CSF Aβ42/40, which does reflect amyloid, and values can reflect non-Alzheimer’s disease factors, including cerebral amyloid angiopathy (CAA)^68,69^ and certain medications.^70^ Therefore, while the relationships of plasma Aβ42/40 with FDG-PET and volumes aligned with neuropathological expectations, no further interpretation is proposed.

### Study limitations

There are several limitations to this study, including the small sample size. This, and the exploratory nature of this study, led us not to apply any correction for multiple comparisons, which increases the likelihood of type I errors, especially for marginally significant results. These type I errors may explain the unexpected positive associations between GFAP and hippocampal volumes. These findings should be replicated in larger, more diverse cohorts to further validate the associations observed here. The blood samples were acquired, processed, and stored before more recent guidelines were developed regarding sample preparation for plasma assays. Amyloid- and tau-PET images were not acquired, which could further elucidate relationships between plasma biomarkers and the downstream functional effects in FDG-PET and volume that may be driven by progression of the pathology.

### Summary and future directions

This exploratory study supports the association of p-tau217 with downstream functional and neurodegenerative processes associated with neurofibrillary tangle spread in Alzheimer’s disease. Our FDG-PET and volumetric findings are consistent with published relationships between plasma p-tau217 and tau-PET, but extended our knowledge by examining neuronal function and volume loss. Results illustrate that nearly identical plasma p-tau217 levels can be associated with diverse spatial distributions and severities of functional and tissue loss. Combining plasma biomarker information with imaging can provide insight into patient status and contributors to decline. Future directions include an investigation of the relationships between FDG-PET, volume, and emerging new plasma tau biomarkers such as MTBR-tau243 that may correlate even more specifically with tau-PET neurofibrillary tangles.^25^

## Supplementary Material

fcag074_Supplementary_Data

## Data Availability

Anonymized data will be shared by request from a qualified academic investigator for the sole purpose of replicating procedures and results presented in the article, and as long as data transfer is in agreement with the IRB of the involved institutions, which should be regulated in a material transfer agreement. Code that was generated for these analyses is available at https://github.com/lnkoenig/ptau-217-and-FDG-PET-AD—official-copy.

## References

[1] DeTure MA, Dickson DW. The neuropathological diagnosis of Alzheimer’s disease. Mol Neurodegener. 2019;14(1):32. 31375134 10.1186/s13024-019-0333-5 PMC6679484

[2] Kakkar A, Singh H, Singh BK, Kumar A, Mishra AK, Chopra H. Neuroinflammation and Alzheimer’s disease: Unravelling the molecular mechanisms. J Alzheimers Dis. 2025;108(1):19–41. 40938771 10.1177/13872877251374353

[3] Nakamura A, Kaneko N, Villemagne VL, et al High performance plasma amyloid-β biomarkers for Alzheimer’s disease. Nature. 2018;554(7691):249–254. 29420472 10.1038/nature25456

[4] Pereira JB, Janelidze S, Smith R, et al Plasma GFAP is an early marker of amyloid-β but not tau pathology in Alzheimer’s disease. Brain. 2021;144(11):3505–3516. 34259835 10.1093/brain/awab223 PMC8677538

[5] Karikari TK, Ashton NJ, Brinkmalm G, et al Blood phospho-tau in Alzheimer disease: Analysis, interpretation, and clinical utility. Nat Rev Neurol. 2022;18(7):400–418. 35585226 10.1038/s41582-022-00665-2

[6] Ashton NJ, Janelidze S, Al Khleifat A, et al A multicentre validation study of the diagnostic value of plasma neurofilament light. Nat Commun. 2021;12(1):3400. 34099648 10.1038/s41467-021-23620-z PMC8185001

[7] Tanne JH . FDA approves blood test to diagnose Alzheimer’s. BMJ. 2025;389:r1082. 40409786 10.1136/bmj.r1082

[8] Ashton NJ, Janelidze S, Mattsson-Carlgren N, et al Differential roles of Aβ42/40, p-tau231 and p-tau217 for Alzheimer’s trial selection and disease monitoring. Nat Med. 2022;28(12):2555–2562. 36456833 10.1038/s41591-022-02074-w PMC9800279

[9] Barthélemy NR, Salvadó G, Schindler SE, et al Highly accurate blood test for Alzheimer’s disease is similar or superior to clinical cerebrospinal fluid tests. Nat Med. 2024;30(4):1085–1095. 38382645 10.1038/s41591-024-02869-z PMC11031399

[10] Mielke MM, Dage JL, Frank RD, et al Performance of plasma phosphorylated tau 181 and 217 in the community. Nat Med. 2022;28(7):1398–1405. 35618838 10.1038/s41591-022-01822-2 PMC9329262

[11] Palmqvist S, Janelidze S, Quiroz YT, et al Discriminative accuracy of plasma phospho-tau217 for Alzheimer disease vs other neurodegenerative disorders. JAMA. 2020;324(8):772–781. 32722745 10.1001/jama.2020.12134 PMC7388060

[12] Cicognola C, Janelidze S, Hertze J, et al Plasma glial fibrillary acidic protein detects Alzheimer pathology and predicts future conversion to Alzheimer dementia in patients with mild cognitive impairment. Alzheimers Res Ther. 2021;13(1):68. 33773595 10.1186/s13195-021-00804-9 PMC8005231

[13] Matthews DC, Kinney JW, Ritter A, et al Relationships between plasma biomarkers, tau PET, FDG PET, and volumetric MRI in mild to moderate Alzheimer’s disease patients. Alzheimers Dement Transl Res Clin Intervent. 2024;10(3):e12490. 10.1002/trc2.12490 PMC11233274 38988416

[14] Mundada NS, Rojas JC, Vandevrede L, et al Head-to-head comparison between plasma p-tau217 and flortaucipir-PET in amyloid-positive patients with cognitive impairment. Alzheimers Res Ther. 2023;15(1):157. 37740209 10.1186/s13195-023-01302-w PMC10517500

[15] Lantero Rodriguez J, Karikari TK, Suárez-Calvet M, et al Plasma p-tau181 accurately predicts Alzheimer’s disease pathology at least 8 years prior to post-mortem and improves the clinical characterisation of cognitive decline. Acta Neuropathol. 2020;140(3):267–278. 32720099 10.1007/s00401-020-02195-x PMC7423866

[16] Salvadó G, Ossenkoppele R, Ashton NJ, et al Specific associations between plasma biomarkers and postmortem amyloid plaque and tau tangle loads. EMBO Mol Med. 2023;15(5):e17123. 36912178 10.15252/emmm.202217123 PMC10165361

[17] Janelidze S, Bali D, Ashton NJ, et al Head-to-head comparison of 10 plasma phospho-tau assays in prodromal Alzheimer’s disease. Brain. 2023;146(4):1592–1601. 36087307 10.1093/brain/awac333 PMC10115176

[18] Lai R, Li B, Bishnoi R. P-tau217 as a reliable blood-based marker of Alzheimer’s disease. Biomedicines. 2024;12(8):1836. 39200300 10.3390/biomedicines12081836 PMC11351463

[19] Schindler SE, Petersen KK, Saef B, et al Head-to-head comparison of leading blood tests for Alzheimer’s disease pathology. Alzheimers Dement. 2024;20(11):8074–8096. 39394841 10.1002/alz.14315 PMC11567821

[20] Ashton NJ, Brum WS, Di Molfetta G, et al Diagnostic accuracy of a plasma phosphorylated tau 217 immunoassay for Alzheimer disease pathology. JAMA Neurol. 2024;81(3):255–263. 38252443 10.1001/jamaneurol.2023.5319 PMC10804282

[21] Devanarayan V, Doherty T, Charil A, et al Plasma pTau217 predicts continuous brain amyloid levels in preclinical and early Alzheimer’s disease. Alzheimers Dement. 2024;20(8):5617–5628. 38940656 10.1002/alz.14073 PMC11350129

[22] Mendes AJ, Ribaldi F, Lathuiliere A, et al Head-to-head study of diagnostic accuracy of plasma and cerebrospinal fluid p-tau217 versus p-tau181 and p-tau231 in a memory clinic cohort. J Neurol. 2024;271(4):2053–2066. 38195896 10.1007/s00415-023-12148-5 PMC10972950

[23] Mattsson-Carlgren N, Janelidze S, Bateman RJ, et al Soluble P-tau217 reflects amyloid and tau pathology and mediates the association of amyloid with tau. EMBO Mol Med. 2021;13(6):e14022. 33949133 10.15252/emmm.202114022 PMC8185545

[24] Therriault J, Ashton NJ, Pola I, et al Comparison of two plasma p-tau217 assays to detect and monitor Alzheimer’s pathology. EBioMedicine. 2024;102:105046. 38471397 10.1016/j.ebiom.2024.105046 PMC10943661

[25] Horie K, Salvadó G, Koppisetti RK, et al Plasma MTBR-tau243 biomarker identifies tau tangle pathology in Alzheimer’s disease. Nat Med. 2025;31(6):2044–2053. 40164726 10.1038/s41591-025-03617-7 PMC12176612

[26] Groot C, Cicognola C, Bali D, et al Diagnostic and prognostic performance to detect Alzheimer’s disease and clinical progression of a novel assay for plasma p-tau217. Alzheimers Res Ther. 2022;14:67. 35568889 10.1186/s13195-022-01005-8 PMC9107269

[27] Khosravi M, Peter J, Wintering NA, et al 18F-FDG is a superior indicator of cognitive performance compared to 18F-florbetapir in Alzheimer’s disease and mild cognitive impairment evaluation: A global quantitative analysis. J Alzheimers Dis. 2019;70(4):1197–1207. 31322568 10.3233/JAD-190220

[28] Landau SM, Harvey D, Madison CM, et al Associations between cognitive, functional, and FDG-PET measures of decline in AD and MCI. Neurobiol Aging. 2011;32(7):1207–1218. 19660834 10.1016/j.neurobiolaging.2009.07.002 PMC2891865

[29] Minoshima S, Cross D, Thientunyakit T, Foster NL, Drzezga A. 18F-FDG PET imaging in neurodegenerative dementing disorders: Insights into subtype classification, emerging disease categories, and mixed dementia with copathologies. J Nucl Med. 2022;63(Suppl 1):2S–12S. 35649653 10.2967/jnumed.121.263194

[30] Marcus C, Mena E, Subramaniam RM. Brain PET in the diagnosis of Alzheimer’s disease. Clin Nucl Med. 2014;39(10):e413–e422; quiz e423-426. 25199063 10.1097/RLU.0000000000000547 PMC4332800

[31] Alexander GE, Chen K, Pietrini P, Rapoport SI, Reiman EM. Longitudinal PET evaluation of cerebral metabolic decline in dementia: A potential outcome measure in Alzheimer’s disease treatment studies. Am J Psychiatry. 2002;159(5):738–745. 11986126 10.1176/appi.ajp.159.5.738

[32] Mosconi L, Tsui WH, Herholz K, et al Multicenter standardized 18F-FDG PET diagnosis of mild cognitive impairment, Alzheimer’s disease, and other dementias. J Nucl Med. 2008;49(3):390–398. 18287270 10.2967/jnumed.107.045385 PMC3703818

[33] Adams JN, Lockhart SN, Li L, Jagust WJ. Relationships between tau and glucose metabolism reflect Alzheimer’s disease pathology in cognitively normal older adults. Cerebral Cortex. 2019;29(5):1997–2009. 29912295 10.1093/cercor/bhy078 PMC6458898

[34] Strom A, Iaccarino L, Edwards L, et al Cortical hypometabolism reflects local atrophy and tau pathology in symptomatic Alzheimer’s disease. Brain. 2022;145(2):713–728. 34373896 10.1093/brain/awab294 PMC9014741

[35] Whitwell JL, Graff-Radford J, Tosakulwong N, et al Imaging correlations of tau, amyloid, metabolism, and atrophy in typical and atypical Alzheimer’s disease. Alzheimers Dement. 2018;14(8):1005–1014. 29605222 10.1016/j.jalz.2018.02.020 PMC6097955

[36] Kadekaro M, Crane AM, Sokoloff L. Differential effects of electrical stimulation of sciatic nerve on metabolic activity in spinal cord and dorsal root ganglion in the rat. Proc Natl Acad Sci U S A. 1985;82(17):6010–6013. 3862113 10.1073/pnas.82.17.6010 PMC390684

[37] Sokoloff L . The physiological and biochemical bases of functional brain imaging. Cogn Neurodyn. 2008;2(1):1–5. 19003468 10.1007/s11571-007-9033-x PMC2289249

[38] La Joie R, Visani AV, Baker SL, et al Prospective longitudinal atrophy in Alzheimer’s disease correlates with the intensity and topography of baseline tau-PET. Sci Transl Med. 2020;12(524):eaau5732. 31894103 10.1126/scitranslmed.aau5732 PMC7035952

[39] Matthews DC, Ritter A, Thomas RG, et al Rasagiline effects on glucose metabolism, cognition, and tau in Alzheimer’s dementia. Alzheimers Dement (N Y). 2021;7(1):e12106. 33614888 10.1002/trc2.12106 PMC7882538

[40] Ossenkoppele R, Schonhaut DR, Schöll M, et al Tau PET patterns mirror clinical and neuroanatomical variability in Alzheimer’s disease. Brain. 2016;139(Pt 5):1551–1567. 26962052 10.1093/brain/aww027 PMC5006248

[41] Matthews DC, Mao X, Dowd K, et al Riluzole, a glutamate modulator, slows cerebral glucose metabolism decline in patients with Alzheimer’s disease. Brain. 2021;144(12):3742–3755. 34145880 10.1093/brain/awab222 PMC8719848

[42] Ashton NJ, Pascoal TA, Karikari TK, et al Plasma p-tau231: A new biomarker for incipient Alzheimer’s disease pathology. Acta Neuropathol. 2021;141(5):709–724. 33585983 10.1007/s00401-021-02275-6 PMC8043944

[43] Karikari TK, Pascoal TA, Ashton NJ, et al Blood phosphorylated tau 181 as a biomarker for Alzheimer’s disease: A diagnostic performance and prediction modelling study using data from four prospective cohorts. Lancet Neurol. 2020;19(5):422–433. 32333900 10.1016/S1474-4422(20)30071-5

[44] Seabold S, Perktold J. Statsmodels: Econometric and statistical modeling with python. SciPy. 2010.

[45] Aziz AL, Giusiano B, Joubert S, et al Difference in imaging biomarkers of neurodegeneration between early and late-onset amnestic Alzheimer’s disease. Neurobiol Aging. 2017;54:22–30. 28314160 10.1016/j.neurobiolaging.2017.02.010

[46] Fujishima M, Kawasaki Y, Mitsuhashi T, Matsuda H, For the Alzheimer’s Disease Neuroimaging Initiative. Impact of amyloid and tau positivity on longitudinal brain atrophy in cognitively normal individuals. Alzheimers Res Ther. 2024;16(1):77. 38600602 10.1186/s13195-024-01450-7 PMC11005141

[47] Ishibashi K, Onishi A, Fujiwara Y, Oda K, Ishiwata K, Ishii K. Longitudinal effects of aging on 18F-FDG distribution in cognitively normal elderly individuals. Sci Rep. 2018;8(1):11557. 30068919 10.1038/s41598-018-29937-y PMC6070529

[48] Lewczuk P, Ermann N, Andreasson U, et al Plasma neurofilament light as a potential biomarker of neurodegeneration in Alzheimer’s disease. Alzheimers Res Ther. 2018;10(1):71. 30055655 10.1186/s13195-018-0404-9 PMC6064615

[49] Moscoso A, Grothe MJ, Ashton NJ, et al Longitudinal associations of blood phosphorylated Tau181 and neurofilament light chain with neurodegeneration in Alzheimer disease. JAMA Neurol. 2021;78(4):396–406. 33427873 10.1001/jamaneurol.2020.4986 PMC7802009

[50] Thijssen EH, Joie RL, Strom A, et al Plasma phosphorylated tau 217 and phosphorylated tau 181 as biomarkers in Alzheimer’s disease and frontotemporal lobar degeneration: A retrospective diagnostic performance study. Lancet Neurol. 2021;20(9):739–752. 34418401 10.1016/S1474-4422(21)00214-3 PMC8711249

[51] Saloner R, VandeVrede L, Asken BM, et al Plasma phosphorylated tau-217 exhibits sex-specific prognostication of cognitive decline and brain atrophy in cognitively unimpaired adults. Alzheimers Dement. 2024;20(1):376–387. 37639492 10.1002/alz.13454 PMC10843677

[52] Strother S, Oder A, Spring R, Grady C. The NPAIRS computational statistics framework for data analysis in neuroimaging. In: Lechevallier, Y., S aporta, G, eds. *Proceedings of COMPSTAT'2010*. Physica-Verlag HD; 2010. 10.1007/978-3-7908-2604-3_10

[53] Ballard C, Atri A, Boneva N, et al Enrichment factors for clinical trials in mild-to-moderate Alzheimer’s disease. Alzheimers Dement. 2019;5:164–174. 10.1016/j.trci.2019.04.001 PMC6527908 31193334

[54] Braak H, Braak E. Neuropathological stageing of Alzheimer-related changes. Acta Neuropathol. 1991;82(4):239–259. 1759558 10.1007/BF00308809

[55] Groot C, Smith R, Collij LE, et al Tau positron emission tomography for predicting dementia in individuals with mild cognitive impairment. JAMA Neurol. 2024;81(8):845–856. 38857029 10.1001/jamaneurol.2024.1612 PMC11165418

[56] Bayoumy S, Verberk IMW, den Dulk B, et al Clinical and analytical comparison of six Simoa assays for plasma P-tau isoforms P-tau181, P-tau217, and P-tau231. Alzheimers Res Ther. 2021;13(1):198. 34863295 10.1186/s13195-021-00939-9 PMC8645090

[57] Ferreira PCL, Brum WS, Ferrari-Souza JP, et al Plasma p-Tau181 and p-Tau231 offer complementary information to identify Alzheimer’s disease pathophysiology. Alzheimer’s & Dementia. 2021;17(S5):e057860.

[58] Ossenkoppele R, Reimand J, Smith R, et al Tau PET correlates with different Alzheimer’s disease-related features compared to CSF and plasma p-tau biomarkers. EMBO Mol Med. 2021;13(8):e14398. 34254442 10.15252/emmm.202114398 PMC8350902

[59] Jarek DJ, Mizerka H, Nuszkiewicz J, Szewczyk-Golec K. Evaluating p-tau217 and p-tau231 as biomarkers for early diagnosis and differentiation of Alzheimer’s disease: A narrative review. Biomedicines. 2024;12(4):786. 38672142 10.3390/biomedicines12040786 PMC11048667

[60] Nagele RG, D’Andrea MR, Lee H, Venkataraman V, Wang HY. Astrocytes accumulate A beta 42 and give rise to astrocytic amyloid plaques in Alzheimer disease brains. Brain Res. 2003;971(2):197–209. 12706236 10.1016/s0006-8993(03)02361-8

[61] Simpson JE, Ince PG, Lace G, et al Astrocyte phenotype in relation to Alzheimer-type pathology in the ageing brain. Neurobiol Aging. 2010;31(4):578–590. 18586353 10.1016/j.neurobiolaging.2008.05.015

[62] Teunissen CE, Verberk IMW, Thijssen EH, et al Blood-based biomarkers for Alzheimer’s disease: Towards clinical implementation. Lancet Neurol. 2022;21(1):66–77. 34838239 10.1016/S1474-4422(21)00361-6

[63] Ally M, Sugarman MA, Zetterberg H, et al Cross-sectional and longitudinal evaluation of plasma glial fibrillary acidic protein to detect and predict clinical syndromes of Alzheimer’s disease. Alzheimers Dement (Amst). 2023;15(4):e12492. 37885919 10.1002/dad2.12492 PMC10599277

[64] Peretti DE, Boccalini C, Ribaldi F, et al Association of glial fibrillary acid protein, Alzheimer’s disease pathology and cognitive decline. Brain. 2024;147(12):4094–4104. 38940331 10.1093/brain/awae211 PMC11629700

[65] Asken BM, VandeVrede L, Rojas JC, et al Lower white matter volume and worse executive functioning reflected in higher levels of plasma GFAP among older adults with and without cognitive impairment. J Int Neuropsychol Soc. 2022;28(6):588–599. 34158138 10.1017/S1355617721000813 PMC8692495

[66] Zhang Y, Wang J, Zhang H, et al Elevated circulating levels of GFAP associated with reduced volumes in hippocampal subregions linked to mild cognitive impairment among community-dwelling elderly individuals. Front Aging Neurosci. 2024;16:1461556. 39534430 10.3389/fnagi.2024.1461556 PMC11554497

[67] Motta C, Di Donna MG, Bonomi CG, et al Different associations between amyloid-βeta 42, amyloid-βeta 40, and amyloid-βeta 42/40 with soluble phosphorylated-tau and disease burden in Alzheimer’s disease: A cerebrospinal fluid and fluorodeoxyglucose-positron emission tomography study. Alzheimers Res Ther. 2023;15(1):144. 37649105 10.1186/s13195-023-01291-w PMC10466826

[68] Hsieh PF, Tsai HH, Liu CJ, et al Plasma phosphorylated tau 217 as a discriminative biomarker for cerebral amyloid angiopathy. Eur J Neurol. 2025;32(2):e70066. 39907306 10.1111/ene.70066 PMC11795418

[69] Muir RT, Stukas S, Cooper JG, et al Plasma biomarkers distinguish Boston Criteria 2.0 cerebral amyloid angiopathy from healthy controls. Alzheimers Dement. 2025;21(3):e70010. 40156276 10.1002/alz.70010 PMC11953569

[70] Blasko I, Jungwirth S, Jellinger K, et al Effects of medications on plasma amyloid beta (abeta) 42: Longitudinal data from the VITA cohort. J Psychiatr Res. 2008;42(11):946–955. 18155247 10.1016/j.jpsychires.2007.10.010

