# Supplementary material for: Plasma p-tau217 and glucose metabolism correlate in neocortical association areas in Alzheimer's disease
Source: Brain Commun. 2026 Mar 9;8(2):fcag074. doi: 10.1093/braincomms/fcag074 (PMC13070614; doi:10.1093/braincomms/fcag074)
Supplement: fcag074_Supplementary_Data [file fcag074_supplementary_data.zip › Supplementary_material.pdf]

Table of Contents

Supplementary Table 1: Full Model Results.. 2

Supplementary Text 1: Analyses when repeated with alternative FDG-PET reference region (Pons) 3

Supplementary Text 2: Analyses when GFAP and NfL Are Adjusted for Normal Aging 4

Supplementary Figure 1: Example FDG And Volumetric Images 5

Supplementary Figure 2: Random Sampling Distributions of Significant Models....................... 7

Supplementary Table 2: Select FDG and Volume Relationships... 8

Supplementary Figure 3: P-tau Interrelationships 9

Supplementary Figure 4: Medial Temporal Lobe FDG & p-tau181 and p-tau231 relationships .10

Supplementary Table 3: Medial Temporal Lobe FDG & p-tau181 relationships Within Subgroups 12

Supplementary Table 1: Full Model Results

See separately included excel file.

Supplementary Text 1: Analyses when repeated with alternative FDG-PET reference region (Pons)

When the analyses were repeated using FDG-PET SUVRs normalized to the pons instead of our paracentral region, few changes in the results were seen. No models were significant in only the pons-normalized data, and only a few of our reported significant relationships lost significance when using the pons reference region. Specifically, plasma p-tau217 and precuneus FDG-PET were no longer significant (β = -0.21, P = 0.24). Plasma Aβ42/40 was no longer related to Medial Temporal FDG-PET (β = 0.11, P = 0.54) nor to Precuneus FDG-PET (β = 0.20, P = 0.27). Plasma p-tau181 and p-tau231 were no longer related to Medial Temporal FDG-PET (β = 0.27, P = 0.10; β = 0.25, P = 0.16, respectively), nor was plasma p-tau181 related to Posterior Cingulate FDG-PET (β = 0.16, P = 0.28).

Supplementary Text 2: Analyses when GFAP and NfL Are Adjusted for Normal Aging

An in-house dataset of 21 healthy controls age 57-80 were used to estimate normal aging effects for plasma GFAP and NfL. The subjects had a similar age range as the Alzheimer disease participants in this study and had plasma taken and measured using the same assay. When modeled, this cohort indicated NFL increased an average of 0.91 pg/mL per year and GFAP increased an average of 3.48 pg/mL per year. These results were used to adjust the Alzheimer disease participant's GFAP and NfL data to remove the effects of normal aging.

When the age-adjusted NfL data was used, plasma NfL (log10) lost significance for its relationship to right putamen volume z-score ($\beta = -0.34$, $P = 0.06$) and gained significance for its relationship to Controlled Oral Word Association Test (COWAT) ($\beta = -0.33$, $P = 0.046$) and Plasma P-tau231 (log10) ($\beta = 0.39$, $P = 0.01$).

When the age-adjusted data GFAP was used, plasma GFAP (log10) lost significance for Left Angular Gyrus Volume z-score ($\beta = -0.29$, $P = 0.06$) and gained significance for Left Superior Frontal Volume z-score ($\beta = -0.51$, $P = 0.003$) and Left Supramarginal Gyrus Volume z-score ($\beta = -0.35$, $P = 0.04$). Additionally, GFAP gained significance for CDR Sum of Boxes ($\beta = 0.37$, $P = 0.03$), Inferior Parietal FDG-PET SUVR ($\beta = -0.32$, $P = 0.04$), and Precuneus FDG-PET SUVR ($\beta = -0.35$, $P = 0.02$).

Supplementary Figure 1: Example FDG And Volumetric Images

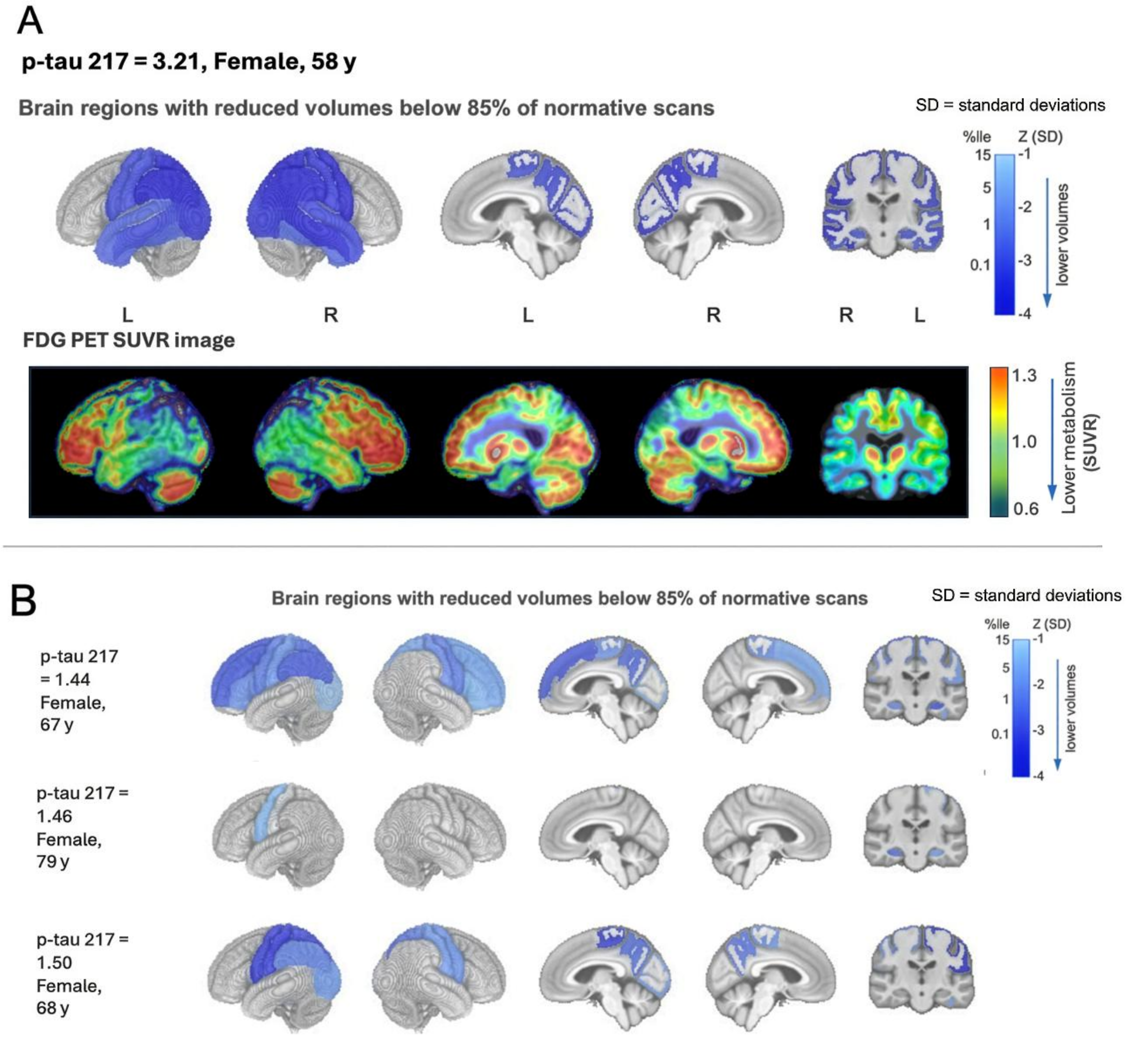

Supplementary Figure 1 Example FDG-PET and volumetric images. (A) Example of a participant with high p-tau217 value and atrophy and hypometabolism in regions including temporal and parietal cortices as well as para and post central gyri. Regions with reduced volumes are shown with blue shading, by age- and sex- matched z-score relative to normative data. The FDG-PET image is shown in the second row, color coded for SUVR. Participants having effects in para, post, and/or precentral regions may reflect late Braak stages of tau accumulation and can also be found among people exhibiting an amyloid positive corticobasal degeneration-like pattern.

(B) Examples of participants with very similar p-tau217 values, but distinct spatial atrophy effects. Abbreviations: FDG-PET = [18F]Fluorodeoxyglucose positron emission tomography, SUVR = Standardized Uptake Value Ratio.

Supplementary Figure 2: Random Sampling Distributions of Significant Models

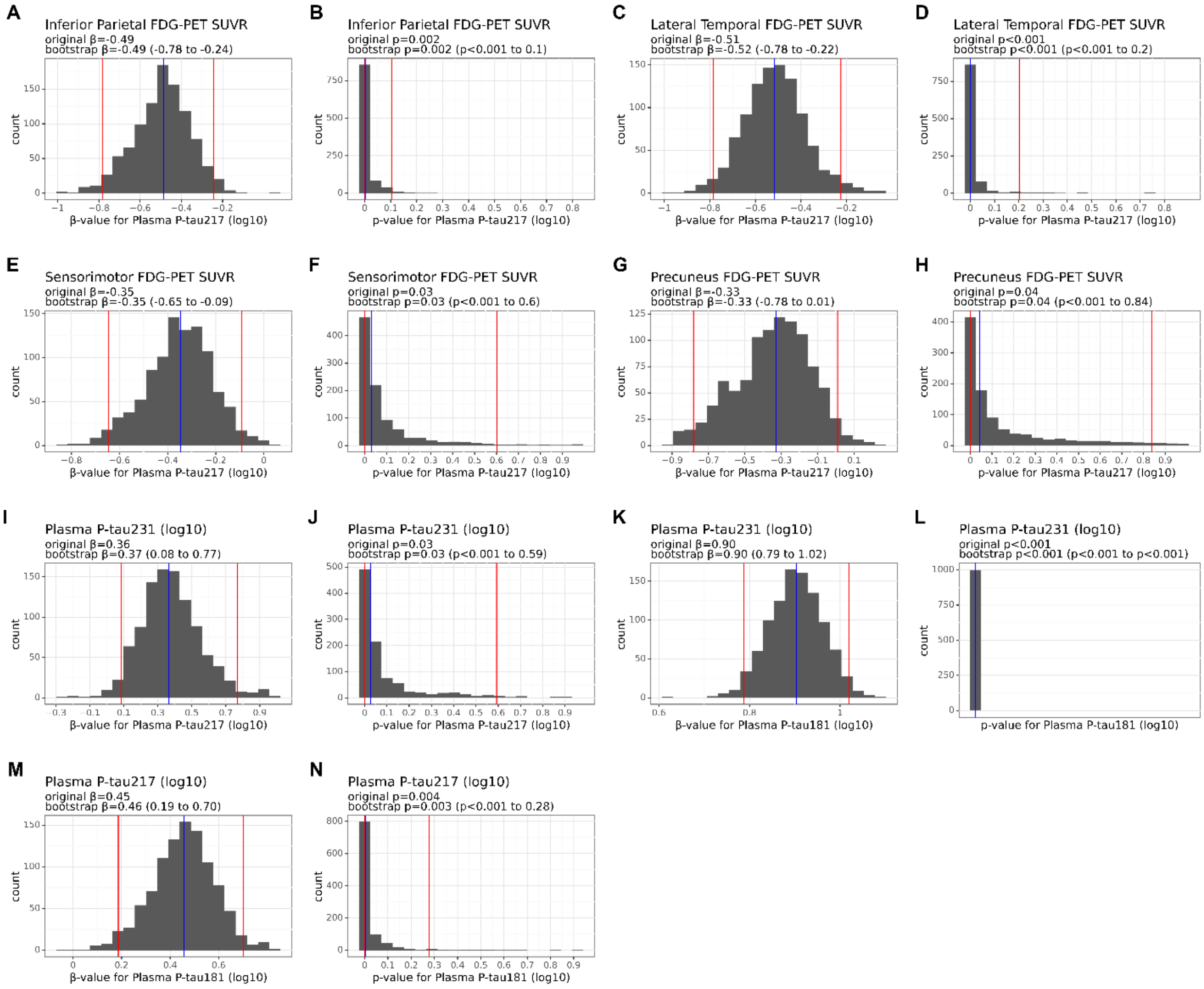

Supplementary Figure 2 Random sampling distribution of significant models. Displays how some of our key results vary across 1000 bootstrap replicates. The blue vertical lines on the graphs mark the median *P-value* while the red lines mark 95% confidence intervals. Alongside the original *P-value* (that from the actual data), the median and 95% confidence interval from the 1000 bootstrap replicates gives a sense of the consistency and strength of our results, given the small sample size. Abbreviations: FDG-PET = [18F]Fluorodeoxyglucose positron emission tomography, SUVR = Standardized Uptake Value Ratio.

Supplementary Table 2: Select FDG and Volume Relationships

Y Variable	X Variable	# of participants	X variable β-value	X variable P-value	Intercept β-value	Intercept P-value
Left Inferior Parietal Volume z-score	Inferior Parietal FDG-PET SUVR	42	0.59	<0.001	0.00	1.0
Right Inferior Parietal Volume z-score	Inferior Parietal FDG-PET SUVR	42	0.55	<0.001	0.00	1.0

Abbreviations: FDG-PET = [18F]Fluorodeoxyglucose Positron Emission Tomography, SUVR = standardized uptake value ratios

Supplementary Figure 3: P-tau Interrelationships

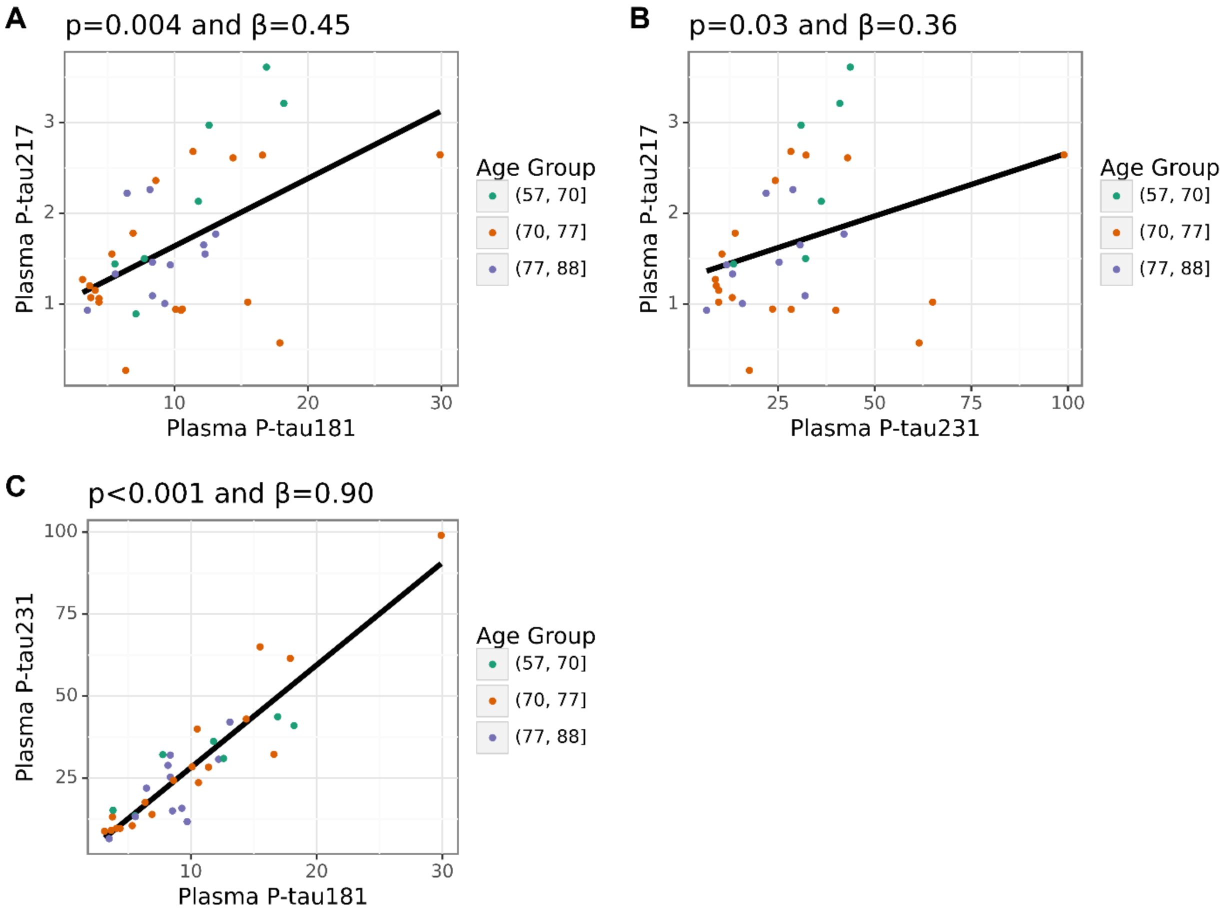

Supplementary Figure 3 P-tau interrelationships. Supplementary Figure 3 displays how the three measured plasma p-tau markers (p-tau217, p-tau231, and p-tau181) relate to each other in this cohort. Individual participants' points are colored based on age to reveal any age-specific relationships, a black trend line is included to help to indicate the relationship, and plasma p-tau is displayed in its raw (not log-10-transformed) format. The *P-value* and *β*-value displayed in the title for each subgraph are the results of the main analyses, which are fully described in Supplemental Table 1. These analyses used log-10-transformed plasma data in a general linear model and included covariates if they were significant. The graphs show the actual data points without log transformation for the purposes of visualization.

Supplementary Figure 4: Medial Temporal Lobe FDG & p-tau181 and p-tau231 relationships

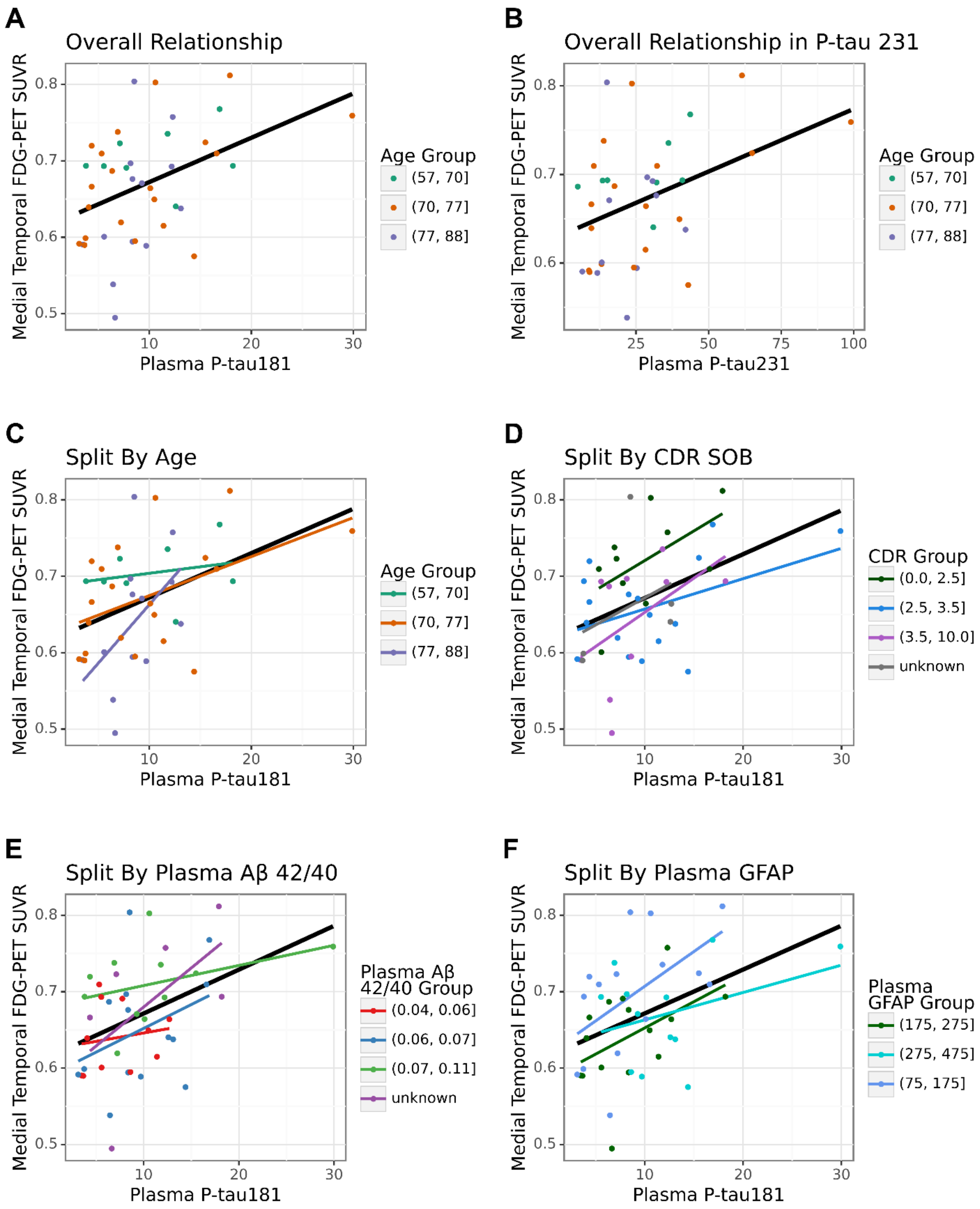

Supplementary Figure 4 Medial temporal lobe FDG relationships with p-tau181 and p-tau231. Supplementary Figure 4 displays our investigation into the relationship of Medial Temporal FDG-PET with plasma p-tau181 and with plasma p-tau231, which were not in the expected direction ($\beta = 0.55$, $P < 0.001$; $\beta = 0.35$, $P = 0.03$, respectively) in the main analyses (Supplemental Table 1). Subplots A and B display the relationship of plasma p-tau181 and p-tau231 with Medial Temporal FDG-PET as was described in the main text, with plasma displayed in its raw (not log-10-transformed) format. We fit additional general linear models of the same type to various subgroups of the cohort to explore possible causes of this unexpected relationship. Subplots C-F display the same individual data points and main relationship seen in subplot A but additionally display the linear fits of the several subgroups. All of the subgroups - age groups, level of impairment (CDR sum of boxes), plasma Aβ levels, and plasma GFAP levels – are directionally consistent with the overall relationship. Though we compared groups only visually due to the small number of subjects in each, the model statistics for these subgroups can be found in Supplementary Table 3. Abbreviations: Aβ = amyloid-beta, CDR SOB = Clinical Dementia Rating scale Sum of Boxes, FDG-PET = [18F]Fluorodeoxyglucose positron emission tomography, GFAP = Glial fibrillary acidic protein, SUVR = Standardized Uptake Value Ratio.

Supplementary Table 3: Medial Temporal Lobe FDG & p-tau181 relationships Within Subgroups

Subgroup Type	Subgroup Label	n	β-value p-Tau181	P-value p-Tau181	β-value Education years	P-value Education years	β-value Intercept	P-value Intercept
age	(57, 70]	8	0.19	0.30	0.39	0.09	0.29	0.16
age	(70, 77]	20	0.43	0.01	0.43	0.04	-0.04	0.85
age	(77, 88]	13	1.22	0.03	0.65	0.04	0.21	0.53
CDR sum of boxes	(0.0, 2.5]	10	0.62	0.13	0.14	0.72	0.62	0.08
CDR sum of boxes	(2.5, 3.5]	17	0.32	0.02	0.45	0.006	-0.17	0.26
CDR sum of boxes	(3.5, 10.0]	10	0.35	0.35	0.72	0.04	-0.08	0.78
CDR sum of boxes	unknown	4	2.21	0.34	1.28	0.37	0.69	0.54
Aβ42/40	(0.04, 0.06]	10	0.37	0.41	0.37	0.17	-0.25	0.46
Aβ42/40	(0.06, 0.07]	14	0.58	0.05	0.63	0.0097	-0.10	0.64
Aβ42/40	(0.07, 0.11]	11	0.19	0.24	0.28	0.19	0.47	0.048
Aβ42/40	unknown	6	0.81	0.23	0.69	0.36	-0.08	0.91
GFAP	(75, 175]	15	0.77	0.01	0.60	0.08	0.30	0.26
GFAP	(175, 275]	14	0.51	0.10	0.31	0.23	-0.26	0.29
GFAP	(275, 475]	12	0.21	0.11	0.57	0.0007	0.15	0.37

Abbreviations: Aβ = beta-amyloid, CDR = Clinical Dementia Rating scale, FDG-PET = [18F]Fluorodeoxyglucose Positron Emission Tomography, GFAP = glial fibrillary acidic protein, SUVR = standardized uptake value ratios
